# Strategies for the transition of learners with specific learning disabilities from remedial to mainstream schooling in South Africa

**DOI:** 10.4102/ajod.v15i0.1900

**Published:** 2026-06-09

**Authors:** Siboniso J. Selepe, Mohamed Mosala, Katlego Tshiloane, Maximus M. Sefotho

**Affiliations:** 1 Department of Educational Psychology, Faculty of Education, University of Johannesburg, Johannesburg, South Africa

**Keywords:** inclusion, transitioning, specific learning disabilities, individual support plan, assistive technology

## Abstract

**Background:**

Education involves recognising the dignity and learning potential of every learner. This study examines the ethical and practical meaning of *transitioning* learners with specific learning disabilities (SLD) from remedial to mainstream schooling within South Africa’s inclusive education policy framework.

**Objectives:**

This study explores philosophical and practical strategies that support effective transitioning of learners with SLD into mainstream schools.

**Method:**

A qualitative single-case study design was employed involving seven participants in Johannesburg. Data were collected through semi-structured interviews, reflective journals and document analysis, and analysed using thematic analysis guided by Creswell’s analytic spiral.

**Results:**

The results of this study indicate that most teachers lacked formal training in inclusive education despite supporting its principles. Individual support plans (ISPs) help personalise learning through collaboration between teachers, parents and support teams. Assistive technologies and accommodations, like readers and extra time, support equitable learning access. Peer support and differentiated instruction enhance confidence, inclusion and engagement for learners with SLD.

**Conclusion:**

The study concludes that successful transitioning of learners with SLD depends on structured support, ethical collaboration and compassionate teaching practices. It calls for stronger alignment between inclusive education policies and classroom realities to ensure every learner’s dignity and belonging.

**Contribution:**

The study contributes by bridging philosophical ideals with practical strategies, offering a framework for inclusive and humane educational practice.

## Introduction

Education, at its deepest level, is not the transmission of information but the awakening of the human spirit. It is the quiet yet radical act of affirming that every person, regardless of difference or difficulty, carries within them an irreplaceable spark of meaning (Mayseless & Kizel 2022). To *transition* a learner with a specific learning disability (SLD) from a remedial school into the mainstream school represents more than an administrative process. It reflects the inclusive education principle that mainstream schooling is the default placement supported through structured educational provisioning (Department of Basic Education 2014). It is a moral and spiritual encounter with the essence of what it means to be human (Huda & Salem 2022). This study unfolds within that sacred space where philosophy and education meet: The space where society must decide whether it will treat difference as a problem to be fixed or as a dimension of beauty to be embraced. To transition is to restore belonging; it is to say, with conviction, that no learner is expendable, that no mind is too divergent to share the common light of understanding.

The philosophical foundations of inclusion are ancient yet urgent. From Aristotle’s (1999) belief in the potentiality of every human being, to Rousseau’s (1979) insistence that education must be shaped by the learner’s nature, to Dewey’s (1916) conviction that democracy begins in the classroom, the tradition of educational philosophy has always carried a prophetic message: Education must serve human wholeness. Dewey (1916) viewed education as how individuals grow into participation with others, a living dialogue between self and society. Freire (2009), in his *Pedagogy of the Oppressed*, called education an act of love and courage, a means of transforming both the learner and the world. Nussbaum’s (2011) *capabilities approach* reminds us that education should expand what each person is able to do and that freedom should be realised in action. Within these traditions, transitioning is not a mechanical return but a re-entry into the moral community of learning.

Philosophically, the learner with SLD stands as a mirror reflecting the system’s own humanity. The challenge is not only to teach the learner but to ask whether the school itself can learn, to rethink its definitions of intelligence, progress and success. Policies like South Africa’s *Screening, Identification, Assessment and Support* (SIAS) framework and *Education White Paper 6* carry within them this deeper philosophical promise: That the worth of a school is measured by the dignity it affords its most vulnerable members (Department of Basic Education 2014; Department of Education 2001). To transition, then, is to enact the philosophy of Ubuntu: *I am because we are* (Maphalala & Nkosi 2025). It is to affirm that learning happens not through isolation, but through relationship; that the classroom becomes whole only when every voice, however soft, is heard.

Within the South African inclusive education system, the SIAS policy provides the operational mechanism through which schools identify learning barriers, design individual support plans (ISPs) and coordinate support across school-based support teams (SBST) and district-based support teams (DBST). In practice, these policies guide teachers to document support strategies, monitor learner progress and adapt teaching approaches to ensure participation and progression within mainstream classrooms.

Despite global and national commitments to inclusion, a disquieting gap remains between principle and lived experience. The discourse of *support* and *intervention* often carries subtle echoes of deficit thinking, an assumption that the learner must be repaired before being welcomed back. Such language, though well-intentioned, risks stripping education of its moral power. The philosophical dilemma is this: How can education honour the equality of all learners while remaining faithful to the uniqueness of each? True inclusion does not erase difference; it dignifies it. Transitioning must therefore be reimagined not as assimilation into the normal, but as transformation of the ordinary. This study invites teachers, policymakers, and scholars to pause and ask: What vision of the learner guides our practices? Do we see the child with SLD as a bearer of deficit or as a bearer of possibility? Can a school claim to be inclusive if its concept of ‘success’ excludes the slow, the reflective, or the non-linear mind? These questions are not peripheral; they are philosophical imperatives that cut to the core of educational ethics.

This study builds on existing research examining teachers’ experiences of implementing inclusive education in South African full-service schools (Tshiloane & Sefotho 2025), which highlighted challenges such as inadequate training, limited support and difficulties with curriculum adaptation. While that study focused on teachers’ experiences within inclusive education contexts, the present study extends this work by specifically examining the transitioning of learners with SLD from remedial to mainstream schooling, with particular attention to the instructional and institutional strategies that support this process.

### Research questions

From this reflective stance arise questions not merely of practice but of meaning:

- *What conception of personhood underlies the process of reintegrating learners with SLD into mainstream schooling?*
- *How can educational strategies embody the ethical virtues of compassion, justice and recognition?*
- *In what ways can transitioning become a living philosophy that restores dignity and belonging, rather than an administrative procedure?*
- *What does it mean for a school to become truly human, an institution that learns from its learners?*

The objectives of this study are to explore the philosophical foundations that provide moral depth and direction to transitioning practices, as well as to examine how instructional and institutional strategies, such as ISPs, assistive devices, accommodations and peer support, can reflect ethical and existential principles of inclusion. Additionally, the aim is to develop a reflective framework that connects philosophy and practice, guiding teachers toward transitioning that is both compassionate and transformative. This study seeks to contribute to a broader understanding of inclusion as an ethical way of being rather than merely a procedural goal.

This study seeks to reawaken the moral imagination of education. It calls for a paradigm shift from technical inclusion to ethical inclusion, from a focus on compliance to a focus on consciousness. It asks teachers to view each transitioning not as a policy mandate, but as an act of recognition, a moment in which society declares: You belong here. Your way of learning enriches us all. When inclusion is lived as philosophy, it reshapes not only classrooms but communities. It teaches empathy, humility and the profound truth that human beings grow through one another. Transitioning becomes a metaphor for a larger human project, the transitioning of compassion into systems, of wisdom into policy, of soul into schooling. A society that embraces its differently learning members does more than educate, it evolves. It becomes capable of awe, of seeing in each learner a living reminder of the infinite ways intelligence can express itself.

### Conceptual framework

Transitioning learners with SLD from remedial into mainstream schooling represents far more than a logistical transition. It is a profound philosophical act that redefines what it means to learn, to belong and to be human. This process is best understood through a conceptual framework that weaves together *Constructivist, Humanistic, Critical, Ubuntu*, and *Resilience* perspectives. Constructivism and socio-cultural theory remind us that learning is not an isolated cognitive process but a co-constructed journey of meaning-making that unfolds within relationships (Brau 2020; Chirkov 2020). Vygotsky’s zone of proximal development and Tomlinson’s notion of differentiated instruction affirm that understanding is nurtured through collaboration, scaffolding, and recognition of individual learning trajectories (Eun 2019; Setambah et al. 2025). Within transitioning, this perspective demands classrooms that adapt to the learner’s readiness rather than forcing the learner to conform to static norms. Knowledge, in this view, becomes a shared construction that honours the learner’s lived experience and unique way of making sense of the world.

Humanistic and phenomenological theories deepen this understanding by situating education in the domain of empathy, affirmation and dialogue (Qutoshi 2018; Sulaiman & Neviyarni 2021). Rogers’ emphasis on unconditional positive regard and Maslow’s hierarchy of needs underscore that learners thrive when they are seen and valued in their wholeness (Putri et al. 2024). For a learner returning from a remedial environment, affirmation is not a luxury, it is the foundation of re-engagement and self-worth. Phenomenologists like Gadamer view education as an encounter of horizons, where teacher and learner meet in a transformative dialogue that reshapes both (Mansouri et al. 2025). Transitioning thus becomes an ethical exchange (Kaplan & Nussio 2018), a hermeneutic process of rediscovering dignity through mutual understanding (Innocent 2025). Critical pedagogy complements this by framing transitioning as a social justice imperative (Giroux 2021). Freire’s (2009) vision of education as a practice of freedom demands that inclusion must go beyond physical presence to genuine participation and voice. Social justice theorists like Nancy Fraser and Iris Marion Young emphasise that genuine inclusion necessitates both the acknowledgement of differences and the redistribution of opportunities (McKeown 2018; Vincent 2019). This means that providing accommodations, assistive technology, and teacher training should not be seen as acts of charity, but rather as essential steps toward achieving equity.

Anchoring these theories within the *Ubuntu philosophy* and the principle of *Resilience* completes the conceptual framework by grounding it in African humanism and lived strength (Aall & Crocker 2019; Kamga 2018). Ubuntu teaches that *umuntu ngumuntu ngabantu* [I am because we are], positioning transitioning as a communal responsibility rather than an individual struggle (Kamga 2018). It affirms that belonging is not granted by institutional acceptance but by relational care. In this view, teachers, peers and parents form a moral community that sustains the learner’s return through empathy, shared responsibility, and collective growth. Resilience, meanwhile, represents the learner’s capacity to flourish amid challenge, not as a solitary trait but as a relational strength cultivated through support and affirmation (Aall & Crocker 2019). When constructivist pedagogy, humanistic affirmation, social justice, Ubuntu’s ethic of interdependence and resilience converge, transitioning becomes an act of re-humanisation. It transforms education from a system of accommodation into a living practice of solidarity and hope, a process through which both the learner and the community rediscover their shared humanity.

### Conceptualising inclusion and transitioning

Inclusive education is more than an educational reform; it is an ethical and philosophical reorientation of the purpose of schooling. It assumes, as Booth and Ainscow (2000) argue in The Index for Inclusion, that schools must *build community and foster values of respect for all*. Transitioning, specifically the return of learners with SLD from remedial to mainstream schooling, emerges from this moral vision. It is the embodiment of what Nussbaum (2011) describes as the capabilities approach: Education as the expansion of human freedom, dignity and participation.

In this sense, inclusion is not simply about placement; it is about presence, participation and progress. It challenges the deficit model of disability, replacing it with a relational understanding of difference (Gibson et al. 2025). Levinas (1969) provides a philosophical anchor for this shift through his ethics of the Other: The idea that encountering difference is not a disruption but the very ground of ethical responsibility. When a learner with SLD returns to the mainstream classroom, society is invited to re-learn its own humanity, to see learning diversity as a mirror of moral obligation rather than deviation.

Empirically, this conception aligns with the UNESCO Salamanca Statement (1994) and Education for All (UNESCO 2009), which affirm that all children should learn together, wherever possible, and that schools must adapt to meet diverse needs. The South African Education White Paper 6 (Department of Education 2001) and the SIAS Policy (Department of Basic Education 2014) operationalise these principles locally, providing frameworks for identifying barriers to learning and coordinating multi-level support structures.

### Instructional and institutional strategies

#### Individual support plans

Individual Support Plans (ISPs) are formal mechanisms for tailoring education to the learner’s specific needs. The SIAS policy mandates their use, echoing international models of Individualised Education Programmes (IEPs) (Department of Education 2014). Philosophically, the ISP embodies the Kantian principle of treating the learner as an end in themselves, never merely as a means (Department of Basic Education 2014; Sticker & Bakhurst 2021). Practically, ISPs coordinate interventions, adjusted pacing, accommodations and regular reviews that ensure continuity between remedial and mainstream contexts. Research (Engelbrecht & Savolainen 2018; Florian & Black-Hawkins 2013) shows that effective ISPs depend on genuine collaboration between teachers, parents and learners. When co-constructed, they become living documents that reflect shared ethical commitment rather than bureaucratic compliance.

#### Assistive technology and accommodations

Assistive devices and accommodations represent the technological arm of inclusion. From screen readers to speech-to-text software, these tools operationalise the right to equitable participation (Al-Azawei, Serenelli & Lundqvist 2016). The Concessions Procedural Manual (Department of Basic Education 2024) specifies accommodations such as extra time, readers or scribes, affirming that fairness sometimes requires differentiation. Philosophically, this reflects Rawls’ (2016) principle of justice as fairness: Equity is achieved not by sameness, but by responsiveness to difference. When a learner writes on a laptop instead of by hand, the school is not bending rules; it is fulfilling justice.

#### Peer support and differentiated instruction

Peer relationships are among the most potent yet underappreciated tools in transitioning. Studies show that structured peer support fosters academic engagement and social belonging (Torres, Chen & Peixoto 2025). From a philosophical angle, peer support enacts the ethics of interdependence central to Ubuntu and Levinasian thought: The self becomes itself through the face of the Other (Kamga 2018; Levinas 1969). Differentiated instruction complements this relational approach by recognising that learning is plural (Setambah et al. 2025). Visual, auditory and kinaesthetic strategies (Tomlinson 2014) invite learners to encounter knowledge through multiple gateways. In this sense, differentiation becomes a pedagogy of awe; it honours the many ways human minds make meaning.

### Challenges and critiques

Despite the existence of well-established frameworks for transitioning, the process continues to encounter significant challenges. Research conducted by Bornman and Rose (2017) and Walton (2018) reveals critical gaps in several areas, including teacher preparedness, resource allocation and the provision of systemic follow-up support. Many teachers are not sufficiently trained to address the diverse needs of reintegrating learners, which can hinder the effectiveness of their integration into mainstream settings. Additionally, inadequate allocation of resources, such as specialised training programmes and necessary materials, further complicates the situation. Moreover, transitioning is often approached as a singular event rather than a comprehensive, ongoing process that requires consistent attention and adjustment. This perspective can lead to a lack of sustained engagement from both teachers and students, resulting in learners feeling isolated or marginalised in the very spaces intended to foster their inclusion. To create a truly inclusive environment, it is essential to view transitioning as a continuous journey that involves collaboration, support, and a commitment to addressing the unique challenges faced by each learner.

Philosophically, this emphasises a profound contradiction inherent in modern educational practices. The aspiration for equality clashes uneasily with the ever-increasing pressure for standardisation. Hancock (2023) warned that such modern systems may reduce human beings to mere functions within an impersonal machinery, stripping away individuality and personal agency. When the concept of inclusion shifts from a relationship-based approach to a procedural one, students risk becoming invisible, lost within the labyrinth of bureaucratic processes. Consequently, the task at hand extends beyond merely improving educational systems; it also requires a deliberate effort to restore the soul of education. This restoration involves adopting a holistic approach that prioritises personal connections and empathy in learning environments. This aligns with what (Koggel & Orme 2010) describe as an ethic of care, an approach that acknowledges and nurtures the inherent dignity and value of each learner.

## Research methods and design

### Method

A qualitative research approach was adopted to explore and understand the experiences of a learner with SLD transitioning from a remedial to a mainstream school (Lim 2024). This approach allowed for an in-depth exploration of participants’ perspectives and the complex interplay of emotional, academic and systemic factors influencing the transition.

### Design

A single-case study design (Yin 2018) was employed. This design was appropriate for examining the transition within its real-life context, enabling a detailed investigation of the learner’s experiences as well as those of parents, teachers and support teams involved in the process.

### Segmentation

The study’s sample population consisted of seven participants aged between 14 years and 50 years. The learner participant was a 14-year-old male learner in Grade 8 who had recently transitioned from a remedial school into a mainstream secondary school. Additional participants included two parents, one SBST coordinator, one mainstream teacher, one remedial teacher, and one DBST official.

### Data collection method

Multiple data generation methods were used to ensure depth and triangulation of findings: *Semi-structured interviews* were conducted with all participants to explore their experiences, perceptions and emotions regarding the transition. Open-ended questions allowed participants to narrate their experiences freely. Interviews were conducted in person at the participating school, were conducted individually, and lasted between 45 and 60 min. All interviews were audio-recorded with participant consent and later transcribed verbatim to ensure accuracy. *Reflective journals* were maintained by parents and teachers to provide longitudinal insights into the learner’s adjustment over time. Reflective journals were analysed chronologically to identify patterns in the learner’s adjustment process over time. Entries were examined for shifts in emotional, social and academic experiences during the transition period. *Document analysis* included the ISP, psychologist reports and relevant policy documents such as the Department of Education (2001), Department of Basic Education (2014), *South African Schools Act* (1996), *Children’s Act* (2005) and the Basic Education Laws Amendment (BELA) Bill (2024). This provided a contextual and policy framework for interpreting the findings.

### Data analysis

Data analysis followed Creswell’s (2013) Data Analysis Spiral. Initial open coding was conducted manually by reading interview transcripts and journal entries line-by-line to identify meaning units. Codes were then grouped into categories reflecting recurring ideas across participants. These categories were subsequently synthesised into broader themes representing shared experiences within the transition process. Data from interviews, journals, and documents were compared to ensure triangulation. An audit trail of coding decisions and theme development was maintained to strengthen analytic transparency.

### Trustworthiness

To ensure the trustworthiness of the study, several qualitative validation strategies were employed. Credibility was strengthened through data triangulation across interviews, reflective journals, and document analysis. Member checking was conducted informally during follow-up conversations with participants to confirm the accuracy of interpretations. Dependability was supported by maintaining a detailed audit trail documenting analytic decisions and coding processes. Confirmability was enhanced through careful documentation of data sources and reflexive notes during analysis. Transferability was supported by providing detailed descriptions of the research context, participants and support structures within the school system.

### Researcher positionality

The researcher’s professional engagement with inclusive education refers to active involvement in school-based and district-level support processes, including collaboration with teachers, SBST and DBST, as well as experience in implementing and monitoring ISPs for learners experiencing barriers to learning. Recognising the potential influence of prior assumptions is essential in research. This includes the belief that inclusive education is inherently beneficial and that learners with SLD can successfully transition into mainstream schooling with appropriate support. Additionally, it is important to consider that collaborative structures such as SBST and DBST function effectively in facilitating this process. Therefore, reflexive journaling was used throughout the research to document personal reflections and minimise interpretive bias. Regular discussions with the research supervisor further supported critical reflection and analytic rigour.

### Ethical considerations

Ethical clearance was obtained from the University’s Research Ethics Committee (No. SEM2-2024-141). The Gauteng Department of Education also gave permission for me to conduct the study at the intended school. Given that one participant (the learner) was under the age of 15, a formal letter was sent to the parent to obtain informed consent, and assent was obtained from the learner prior to participation. Additionally, to enhance the protection of the participants, an educational psychologist was available to provide psychological support as necessary. A teacher also served as a guardian during the learner’s interview to ensure ethical compliance. All participants were thoroughly informed about the study’s aims and objectives. Anonymity was guaranteed, and participants were made aware that their involvement was entirely voluntary. They were entitled to withdraw from the study at any time without facing any adverse consequences. In addition to parental consent, the learner participant provided informed assent prior to participation. Pseudonyms were used to protect participant identities, and all digital recordings and transcripts were stored on password-protected devices. Data will be securely retained for a period of 5 years in accordance with institutional research ethics guidelines before being permanently deleted.

## Results

The findings indicate that both teachers involved in this study became qualified before inclusive education was formally integrated into teacher education and professional training frameworks in South Africa. As a result, they are teaching students with special educational needs without any formal training in this area. While they support the concept of inclusive education, they feel that external factors beyond their control are preventing them from successfully teaching inclusively. The analysis of the data was guided deductively by pre-existing thematic areas aligned with the instructional and institutional strategies identified in the literature and conceptual framework, namely: Individual Support Plans, Assistive Technology and Accommodations, Peer Support, and Differentiated Instruction. These themes were used as analytical lenses through which participant responses were organised and interpreted.

### Theme 1: Individual support plan

The context of the ISP focuses on supporting learners transitioning back into mainstream education after overcoming challenges. It emphasises the collaboration among stakeholders such as the SBST, DBST, teachers, learners and parents, all working to create a personalised ISP that meets each learner’s unique needs. Key elements include promoting inclusion, fostering collaboration, implementing customised interventions like extra time and assistance and emphasising the essential role of parental involvement in the effective execution of the ISP. The SBST coordinator emphasised the importance of utilising an ISP for learners who are being transitioned into a mainstream school. The coordinator expressed the idea in the following manner:

‘What the support plan usually involves is … we know precisely which intervention to put in. So, we know he needs the extra time, and we know he needs the reader and the scribe … What we do try to do is … interview the learner and the parents … looking for … the suitability of the child’s character and demeanour to the school.’ (SBST Coordinator, female, 38 years old)

The SIAS policy document, published in 2014, features a quote on page 35 that resonates with the statements articulated by the SBST coordinators. This alignment emphasises the congruent vision and objectives shared by both entities regarding the principles of inclusion and support within educational contexts:

Parents/caregivers must play a meaningful role in forming a partnership with the teacher to ensure that the support outlined in the Individual Support Plan is successfully implemented. (Department of Basic Education 2014:35)

The member of the DBST emphasised the necessity of providing comprehensive support to the learner within the framework of the ISP:

‘We have an ISP that we recommend teachers to use, where they provide support to the learner, and then record that support.’ (DBST Member, female, 46 years old)

The quote relates to the ongoing assessment and support strategies outlined in the document for learners with special educational needs. It emphasises the importance of reviewing the ISP to evaluate the effectiveness of interventions and the learner’s progress towards specific goals. This assessment not only measures individual achievements but also guides decisions on transitioning into a mainstream educational environment, ensuring that the learner is prepared and supported throughout the process. The focus on progress and goal attainment reflects a commitment to customised educational approaches that cater to each learner’s unique circumstances:

‘We review the learner’s ISP to assess whether the particular learner achieved a set of goals and objectives. This helps us to determine if the learner is making sufficient progress to transition into a mainstream school.’ (Remedial teacher, female, 34 years old)

The teacher emphasised that the ISP plays a crucial role in the transitioning process. This plan has proven effective for the learner, as it addresses his unique needs and learning styles, which differ from those of his peers. By using this tailored approach, personalised strategies can be implemented to enhance his educational experience and support his growth:

‘The use of an individual support plan has come in handy in supporting this learner since he is different from others.’ (Mainstream teacher, female, 30 years old)

The effective implementation of ISPs is crucial for creating an inclusive educational environment that not only acknowledges but actively addresses the diverse and unique needs of each learner. These plans should be tailored to accommodate various learning styles, disabilities and personal circumstances, ensuring that students receive the appropriate support necessary during their transition back into mainstream schooling. By fostering collaboration among educators, support staff, and families, ISPs can help create a nurturing atmosphere that empowers students to thrive academically and socially, ultimately paving the way for their success in a general school.

### Theme 2: Assistive technology and accommodations

The theme discusses the use of assistive devices and accommodations in education for students with specific learning disorders. It emphasises the collaborative efforts of teachers and support staff in modifying the curriculum and emphasises learner feedback on tools like readers and scribes. The Concessions Procedural Manual outlines eligibility for accommodations, while SBST and DBST play key roles in recommending support and training teachers. The learner also commented on the modification and adaptation of the curriculum as follows:

‘In English, it’s on the computer … everything stays legible … Readers and scribes really help … especially in accounting and EMS [*Economics Management Sciences*] exams … What is there is quite good … Stuff like your scribes, and then the alternative to your scribes, like me writing my exams on the laptop.’ (Learner, male, 14 years old)

The Concessions Procedural Manual for the Assessment and Examination of Learners Who Experience Barriers to Assessment from Grade R to 12, 2024, on pages 33 and 35, supports the provision of accommodations for learners with specific learning disorders. Educators can assist these learners by allowing them to type their answers on a computer rather than using a scribe. Additionally, students with reading impairments because of a specific learning disorder may be granted a reader. For those facing severe difficulties with written expression that affect the speed, fluency and legibility of their handwriting, accommodations for using a scribe are also available. These measures aim to ensure equitable access to learning and assessments:

If a computer is a viable option, it could be used instead of a scribe. This would expand the number of learners who can write in one room. In addition, the use of a computer promotes learner independence. (Department of Basic Education 2024:21)

A learner may be granted the use of a reader if there is a significant discrepancy between the learner’s chronological age and reading age. (Department of Basic Education 2024:18)

‘A learner may be granted the use of a scribe if his/her writing speed is very slow, if the writing is illegible or if the use of a computer is not an option for accommodating the aforementioned two barriers.’ (Department of Basic Education 2024:19)

The SBST coordinator shared her own perspectives on the strategies, which involve identifying specific interventions. For instance, she explained that the learner requires extra time, as well as a reader and a scribe:

‘What the support strategies usually involve is … we know precisely which intervention to put in. So, we know he needs the extra time, and we know he needs the reader and the scribe.’ (SBST Coordinator, female, 38 years old)

The DBST member who shared the same sentiment as the SBST Coordinator and the *South African Schools Act* (SASA) document also emphasised the need to modify and adapt the curriculum. Additionally, the DBST assist schools in applying for accommodations for learners, which may include support from a reader, a scribe, extra time or a separate testing space. DBST also provides training for teachers to become certified readers and scribes. The DBST member supported this by indicating:

‘Then we assist the school in applying for that learner for the accommodations … We make recommendations for accommodations for that learner to be accommodated, possibly with a reader and a scribe, or additional time, or a separate venue … We also train teachers to become certified readers and scribes.’ (DBST Member, female, 46 years old)

The DBST member further stated that:

‘So, then we would request a separate venue, a reader and a scribe, or possibly access to a C pen, whether the family can maybe buy a C pen, or the school can procure a C pen, so the school can maybe allocate a budget.’ (DBST Member, female, 46 years old)

The DBST member briefly described aspects of the training programmes designed for teachers, emphasising the need to modify and adapt the curriculum:

‘And then we provide training on different learning barriers at our SBST trainings.’ (DBST Member, female, 46 years old)

They emphasised the crucial need to educate teachers about various learning barriers, such as dyslexia, ADHD and language difficulties, during SBST training sessions. By doing so, they aim to foster a more comprehensive understanding of individualised support strategies customised to each learner’s unique needs. This training will equip teachers with the knowledge and skills necessary to identify and address these challenges effectively, ultimately enhancing the overall learning experience for all learners.

### Theme 3: Peer support and differentiated instruction

The theme explores the importance of peer support and differentiated instruction for learners with SLD. It emphasises how peer relationships aid transitioning into the school environment, with a parent noting that their child seeks help from friends to catch up on missed work and is accepted for who they are. The learner appreciates this assistance, particularly in reading. A remedial teacher emphasised pairing learners with learning disabilities with supportive peers, which fosters friendships and engagement in extracurricular activities. She supports differentiated instruction tailored to learners’ interests, such as puzzles, and utilises various teaching methods, visual, auditory and kinaesthetic, to enhance learning and retention. This demonstrates how collaborative and customised strategies can significantly benefit these learners. Parent 1, the learner’s parent, emphasised strong peer support as an essential aspect during the transitioning process:

‘Then he would exchange numbers with his friends, so that if he missed any schoolwork or whatever the case may be, he could catch up with them. But the beauty in that is his peers have accepted him in the way he is, so he doesn’t feel like he’s one up, but he’s well aware of his challenges.’ (Parent 1, female, 49 years old)

The learner articulated thoughts that closely echo those of the parent, conveying emotions and perspectives that align closely with the parent’s views. This connection is evident in the way the learner expresses feelings, emphasising a significant emotional resonance between them. The shared sentiments demonstrate a deep understanding and reflection of the learners’ experiences and beliefs:

‘If there’s like a word or something I can’t read, I ask them, like, can you tell me what it says?’ (Learner, male, 14 years old)

The remedial teacher also emphasised the value of peer support in learning. A remedial teacher believes that collaboration among learners enhances their understanding of the material and builds confidence and social skills. By encouraging active participation in study groups and constructive feedback, the remedial teacher fosters a sense of community and shared responsibility in their academic journeys:

‘We also facilitate a peer support programme whereby learners with Specific learning disabilities are paired with those peers who can offer academic and emotional support.’ (Remedial teacher, female, 34 years old)

The mainstream teacher emphasised that peer support is essential for integrated learners facing unique challenges. She explained that encouragement from peers creates an inclusive environment, enhances learning and helps students build valuable social skills. She expressed her idea in the following manner:

‘It really helps if the learner is integrated into the whole school environment, that they have friends; they are doing extracurriculars.’ (Mainstream teacher, female, 30 years old)

The teacher also shared her views on differentiated instruction in the curriculum, emphasising its role in addressing the diverse learning needs of students. She emphasised using varied teaching methods, offering assignment choices and conducting ongoing assessments to enhance engagement and understanding. Here are her main points:

‘He loves puzzles … I have a puzzle of the week … He loves asking about and looking at the puzzle of the week and working on it. Whenever I can, I try to bring a computer science kind of aspect to the lesson.’ (Mainstream teacher, female, 30 years old)

The remedial teacher agreed with the teacher’s statement, emphasising the need to adapt teaching methods to address diverse learners’ learning needs effectively:

‘Visual, auditory, and kinaesthetic teaching methods help with retention.’ (Remedial teacher, female, 34 years old)

The results explain the fundamental role that peer support and differentiated instruction play in the academic success of learners with SLD. The establishment of positive peer relationships not only facilitates the transitioning of these learners into the school environment but also fosters emotional acceptance and collaboration among their peers. Insights from both parents and educators underscore the necessity of tailoring pedagogical strategies to accommodate diverse learning needs. By employing a range of instructional methodologies that resonate with individual interests, teachers can enhance learner engagement and knowledge retention while simultaneously cultivating vital social skills and self-efficacy. The creation of an inclusive and supportive school is essential for the holistic development of learners encountering unique challenges, thereby empowering them to excel both academically and socially.

## Discussion

The study examined strategies for transitioning learners with SLD from remedial to mainstream schools in a South African district. The findings revealed that while teachers support inclusive education, they often lack adequate training and resources to implement it effectively. Three key strategies emerged as central to successful transitioning: ISPs, Assistive Technology and Accommodations, and Peer Support with Differentiated Instruction. Together, these strategies reflect both the philosophical and practical dimensions of inclusion, emphasising collaboration, empathy, and tailored pedagogical approaches.

The results show that ISPs play a critical role in ensuring that learners’ needs are systematically addressed during transitioning. Collaboration between teachers, parents and support teams was identified as essential in developing and monitoring these plans. Assistive technologies and accommodations, such as the use of laptops, readers, and scribes, enabled learners to access the curriculum more equitably. Peer support, combined with differentiated instruction, fostered social belonging and encouraged active participation. Despite these positive practices, challenges persisted, including limited teacher training, insufficient resources and a tendency to view transitioning as a one-time event rather than an ongoing process.

The findings align closely with the principles articulated in the SIAS Policy (Department of Basic Education 2014) and the Education White Paper 6 (2001), both of which emphasise a continuum of support for learners experiencing barriers to learning. The study’s emphasis on collaboration in ISPs resonates with Florian and Black-Hawkins (2013), who argue that inclusive pedagogy thrives on shared responsibility among teachers, parents, and learners. Similarly, the findings reflect Vygotsky’s socio-cultural theory, which situates learning within collaborative relationships (Eun 2019).

The reported use of differentiated instruction and assistive technologies also supports Tomlinson’s (2014) framework, which advocates for diverse teaching strategies that accommodate varied learning profiles. The role of assistive technology observed in this study reinforces Al-Azawei et al. (2016) conclusions that technological tools operationalise the principles of Universal Design for Learning, ensuring equitable participation for all learners. Furthermore, peer support as a transitioning strategy corresponds with Torres et al. (2025), who found that structured peer mentoring enhances motivation and school belonging.

From a philosophical standpoint, the emphasis on relational and ethical inclusion aligns with Freire’s (2009) notion of education as a practice of freedom and with Ubuntu philosophy (Kamga 2018; Maphalala & Nkosi 2025), which situates belonging within community and interdependence. However, the study’s findings diverge from some literature that portrays inclusive education as largely policy-driven and systemically coherent. For instance, while frameworks such as the UNESCO Salamanca Statement (1994) advocate for global inclusivity, this study highlights a gap between policy ideals and classroom realities, echoing the concerns of Bornman and Rose (2017) and Walton (2018) about inadequate teacher preparation and resource allocation in South African schools.

The divergence between inclusive ideals and practical implementation can be attributed to contextual limitations. Teachers in this study obtained their qualifications before inclusive education was formally embedded in teacher education curricula. This lack of preparation explains why they perceive systemic and structural barriers, rather than personal resistance, as the main obstacles to effective inclusion. Such findings illuminate what Hancock (2023) describes as the tension between the humanistic goals of education and the depersonalising effects of standardised systems. The study also suggests that while policies like SIAS offer strong procedural guidance, they often fail to capture the lived moral and emotional dimensions of transitioning.

Philosophers like Levinas (1969) and Nussbaum (2011) remind us that inclusion is fundamentally an ethical act, a response to the Other that affirms human dignity. This moral dimension was evident in the participants’ reflections on empathy, belonging and affirmation. The findings thus extend beyond procedural compliance, showing that successful transitioning depends on cultivating authentic human relationships rather than solely following administrative frameworks. Another point of difference with the literature concerns the framing of resilience. Whereas much of the existing research views resilience as an individual trait, this study positions it as relational, a quality sustained through community support and Ubuntu values. This aligns with Aall and Crocker (2019), who argue that resilience is a shared social capacity rather than a solitary strength.

The results point to transitioning as both a pedagogical and moral enterprise. Practically, the effective use of ISPs, accommodations, and peer support demonstrates that inclusion can succeed when grounded in collaboration and respect for learner diversity. Philosophically, the findings reaffirm that education achieves its fullest purpose when it recognises the learner as an autonomous yet interdependent being. Transitioning, in this sense, becomes an act of re-humanisation, a restoration of belonging and dignity within the community of learning. The integration of constructivist, humanistic and Ubuntu principles within the participants’ experiences suggests that inclusive education must move beyond technical adjustments to embrace ethical consciousness. Teachers, parents and policymakers must see transitioning not as assimilation into the mainstream but as a transformation of the mainstream itself. When schools cultivate empathy, differentiated pedagogy, and moral attentiveness, they enact what Freire (2009) calls *education as an act of love and courage*.

### Limitations of the study

This study was conducted with a small and context-specific sample, which limits the generalisability of the findings, focusing on a single-case study involving one learner with an SLD and six other participants, including parents, teachers and support staff. While this approach provided valuable, in-depth insights, it does not capture the full range of experiences that may occur in different schools, districts or cultural contexts. Additionally, the qualitative nature of the research, which relied on participants’ self-reported experiences through interviews and reflective journals, may be influenced by factors such as memory, perception or social desirability bias, resulting in outcomes that reflect subjective understandings rather than measurable results.

Time constraints further limited the research, as the transitioning process is ongoing but was studied over a relatively short period, restricting the observation of long-term outcomes such as sustained academic progress, emotional adjustment, and social integration; future research with longitudinal designs could provide deeper insights into how support strategies evolve. Moreover, the study was conducted in one educational district, which may differ in resource availability and institutional support from other regions, highlighting how variations in school infrastructure, teacher training, and policy implementation could influence the applicability of the findings. Finally, while multiple perspectives were considered, the absence of input from policymakers or curriculum developers limits the understanding of systemic barriers and policy interpretation, suggesting that future research should incorporate such voices to better connect policy formulation with classroom practice.

## Conclusion

This study examined strategies for reintegrating learners with SLD from remedial to mainstream schools, emphasising the philosophical, pedagogical, and institutional aspects of inclusion. Grounded in a qualitative case study, the research revealed that successful transitioning relies on both structural support and ethical engagement among teachers, parents, learners, and support teams. Key strategies identified include the use of ISPs, the implementation of Assistive Technology and Accommodations, and the promotion of peer support and differentiated instruction, all of which create a foundation for academic success and emotional belonging. ISPs ensure that each learner’s needs are systematically addressed through collaboration, while assistive technologies operationalise fairness, allowing learners to show their abilities without being hindered by disabilities. Peer support and differentiated teaching methods foster social and instructional inclusion, promoting confidence, empathy and shared growth in the classroom.

Philosophically, the study reaffirms that transitioning is a moral act grounded in human dignity and justice, advocating for a redefinition of normality and viewing differences as resources. Despite clear policy frameworks such as the SIAS Policy (2014) and Education White Paper 6 (2001), gaps remain between inclusive ideals and pragmatic realities, highlighting the need for ongoing professional development, resource allocation, and a whole-school commitment to inclusivity. The study contributes to ongoing discussions linking inclusive education to ethical reflection, emphasising that inclusion thrives on empathy and collaboration. For policymakers, it underscores the necessity of aligning educational frameworks with classroom realities; for educators, it demonstrates how compassionate practices supported by structured tools like ISPs can transform transitioning into a renewal process for learners and schools.

## References

[CIT0001] Aall, P. & Crocker, C.A., 2019, ‘Building resilience and social cohesion in conflict’, *Global Policy* 10(S2), 68–75. 10.1111/1758-5899.12681

[CIT0002] Al-Azawei, A., Serenelli, F. & Lundqvist, K., 2016, ‘Universal design for learning (UDL): A content analysis of peer reviewed journals from 2012 to 2015’, *Journal of the Scholarship of Teaching and Learning* 16(3), 39–56. 10.14434/JOSOTL.V16I3.19295

[CIT0003] Aristotle, 1999, *Nicomachean ethics*, transl. W. D. Ross, Batoche Books, Kitchener.

[CIT0004] Booth, T. & Ainscow, M., 2000, *Index for Inclusion: Developing Learning and Participation in Schools*, Centre for Studies on Inclusive Education (CSIE), Bristol.

[CIT0005] Bornman, J. & Rose, J., 2017, *Believe that all can achieve: Increasing classroom participation in learners with special support needs*, 2nd edn., Van Schaik Publishers, Pretoria.

[CIT0006] Brau, B., 2020, ‘Constructivism’, in Oksamytna, K. & Karlsrud, J. (eds.), *United Nations peace operations and international relations theory*, pp. 111–128, Manchester University Press, Manchester.

[CIT0007] Chirkov, V., 2020, ‘An introduction to the theory of sociocultural models’, *Asian Journal of Social Psychology* 23(2), 143–162. 10.1111/AJSP.12381

[CIT0008] Creswell, J.W., 2013, *Research design: Qualitative, quantitative, and mixed methods approaches*, 4th edn., SAGE Publications, Thousand Oaks, CA.

[CIT0009] Department of Basic Education, 2014, *Policy on screening, identification, assessment and support*, viewed 09 October 2025, from https://www.education.gov.za/Portals/0/Documents/Policies/SIAS%20Final%2019%20December%202014.pdf.

[CIT0010] Department of Basic Education, 2024, *Procedural manual for the assessment and examination of learners who experience barriers to assessment from Grade R to 12: Accommodations & concessions*, viewed 29 October 2025, from https://natu.org.za/wp-content/uploads/2024/11/2024-Assessment-Accommodations-and-Concessions-Procedural-manual-2024.pdf.

[CIT0011] Department of Education, 2001, *Education white paper 6*, viewed 12 October 2025, from https://www.gov.za/sites/default/files/gcis_document/201409/educ61.pdf.

[CIT0012] Dewey, J. (1916). *Democracy and education: An introduction to the philosophy of education*, Macmillan Publishing, New York.

[CIT0013] Engelbrecht, P. & Savolainen, H., 2018, ‘A mixed-methods approach to developing an understanding of teachers’ attitudes and their enactment of inclusive education’, *European Journal of Special Needs Education* 33(5), 660–676. 10.1080/08856257.2017.1410327

[CIT0014] Eun, B., 2019, ‘The zone of proximal development as an overarching concept: A framework for synthesizing Vygotsky’s theories’, *Educational Philosophy and Theory* 51(1), 18–30. 10.1080/00131857.2017.1421941

[CIT0015] Florian, L. & Black-Hawkins, K., 2013, ‘Exploring inclusive pedagogy’, *British Educational Research Journal* 37(5), 813–828. 10.1080/01411926.2010.501096

[CIT0016] Freire, P., 2009, ‘Pedagogy of the oppressed’, in M.B. Ramos (ed.), *30th anniversary*, pp. 374–386, Continuum, Abington.

[CIT0017] Gibson, B.E., Rice, C., Gladstone, B.M., Gray, J., Kelly, E., Mosleh, D. et al., 2025, ‘Differences as potentials: A posthuman re-envisioning of disability and mobility’, *Health: An Interdisciplinary Journal for the Social Study of Health, Illness and Medicine* 29(6), 838–856. 10.1177/13634593251329489 PMC12521776 40132133

[CIT0018] Giroux, H., 2021, ‘Critical Pedagogy’, in U. Bauer, U.H. Bittlingmayer & A. Scherr (eds.), *Handbuch Bildungs- und Erziehungssoziologie*, Springer VS, Wiesbaden.

[CIT0019] Hancock, P.A., 2023, ‘Are humans still necessary?’ *Ergonomics* 66(11), 1711–1718. 10.1080/00140139.2023.2236822 37530394

[CIT0020] Huda, M. & Salem, S., 2022, ‘Understanding human behavior development with spirituality: Critical insights into moral flourishing’, *Ulumuna* 26(2), 238–268. 10.20414/ujis.v26i2.535

[CIT0021] Innocent, M.H., 2025, ‘Interreligious dialogue for peacebuilding: A hermeneutical-philosophical approach to the ethics of understanding in a post-conflict Africa’, *Asian Research Journal of Arts & Social Sciences* 23(8), 33–47. 10.9734/arjass/2025/v23i8748

[CIT0022] Kamga, S.D., 2018, ‘Cultural values as a source of law: Emerging trends of ubuntu jurisprudence in South Africa’, *African Human Rights Law Journal* 18(2), 626–649. 10.17159/1996-2096/2018/v18n2a9

[CIT0023] Kaplan, O. & Nussio, E., 2018, ‘Community counts: The social reintegration of ex-combatants in Colombia’, *Conflict Management and Peace Science* 35(2), 132–153. 10.1177/0738894215614506

[CIT0024] Koggel, C. & Orme, J., 2010, ‘Care ethics: New theories and applications’, *Ethics and Social Welfare* 4(2), 109–114. 10.1080/17496535.2010.484255

[CIT0025] Levinas, E., 1969, *Totality and infinity: An essay on exteriority*, in A. Lungis (ed.), Springer/Kluwer, Dordrecht.

[CIT0026] Lim, W.M., 2024, ‘What is qualitative research? An overview and guidelines’, *Australasian Marketing Journal* 33(2), 199–229. 10.1177/14413582241264619

[CIT0027] Mansouri, F., Salahshouri, A., Fayyaz, I., Beheshti, S. & Azadmanesh, S., 2025, ‘Analysis of the meaning of understanding in Gadamer’s perspective to present a conceptual model of teaching-learning strategies’, *Journal of Study and Innovation in Education and Development* 4(5), 263–287. 10.61838/jsied.4.5.14

[CIT0028] Maphalala, M.C. & Nkosi, N., 2025, ‘Fostering ubuntu in teacher education for south African higher education’, *Journal of Education and Learning Technology* 6(1), 10–25. 10.38159/jelt.2025612

[CIT0029] Mayseless, O. & Kizel, A., 2022, ‘Preparing youth for participatory civil society: A call for spiritual, communal, and pluralistic humanism in education with a focus on community of philosophical inquiry’, *International Journal of Educational Research* 115, 102015. 10.1016/j.ijer.2022.102015

[CIT0030] McKeown, M., 2018, ‘Iris marion young’s “social connection model” of responsibility: Clarifying the meaning of connection’, *Journal of Social Philosophy* 49(3), 484–502. 10.1111/JOSP.12253

[CIT0031] Nussbaum, M.C., 2011, *Creating Capabilities: The human development approach*, Belknap Press of Harvard University Press, Cambridge, MA.

[CIT0032] Putri, N.H., Yusuf, A., Prayuga, N.G.A.P. & Syafira, N.P., 2024, ‘Learning theory according to humanistic psychology and its implementation in students’, *Progres Pendidikan* 5(1), 64–70. 10.29303/prospek.v5i1.542

[CIT0033] Qutoshi, S.B., 2018, ‘Phenomenology: A philosophy and method of inquiry’, *Journal of Education and Educational Development* 5(1), 215. 10.22555/JOEED.V5I1.2154

[CIT0034] Rawls, J., 2016, ‘A theory of justice’, in L. May (ed.), *Applied ethics*, 6th edn., pp. 21–28. Routledge, New York, NY.

[CIT0035] Republic of South Africa, 2024, *Basic education laws amendment act 32*, viewed 27 October 2025, from https://www.gov.za/sites/default/files/gcis_document/202409/51258basiceducationlawsamendmentact32of2024.pdf.

[CIT0036] Rousseau, J., 1979, *Emile: Or On Education*, Basic Books, Minnesota.

[CIT0037] Setambah, M.A.B, Appalanaidu, S.R., Zaini, S.H., Othman, M.S, Ridwan, A. & Ibrahim, M.A., 2025, ‘Differentiated instruction: A systematic literature review on implementation guidelines in mathematics education’, *International Journal of Advanced and Applied Sciences* 12(3), 101–108 10.21833/ijaas.2025.03.011

[CIT0038] Sticker, M. & Bakhurst, D., 2021, ‘Kant on education and improvement: Themes and problems’, *Journal of Philosophy of Education* 55(6), 909–920. 10.1111/1467-9752.12631

[CIT0039] Sulaiman, S. & Neviyarni, S., 2021, ‘Teori belajar menurut aliran psikologi humanistik serta implikasinya dalam proses belajar dan pembelajaran [Learning theory according to humanistic psychology and its implications in the learning and teaching process]’, *Jurnal Sikola: Jurnal Kajian Pendidikan dan Pembalajaran* 2(3), 220–234. 10.24036/sikola.v2i3.118

[CIT0040] The Government of South Africa, 1996, *South African Schools Act*, viewed 27 February 2025, from https://www.education.gov.za/LinkClick.aspx?fileticket=aIolZ6UsZ5U%3D&tabid=185&mid=1828.

[CIT0041] The Government of South Africa, 2005, *Children’s Act*, viewed 25 October 2025, from https://www.justice.gov.za/legislation/acts/2005-038%20childrensact.pdf.

[CIT0042] Tomlinson, S., 2014, *The politics of race, class and special education: The selected works of Sally Tomlinson*, 1st edn., Routledge, London.

[CIT0043] Torres, J., Chen, D. & Peixoto, B., 2025, ‘Effect of peer-mentoring programs on academic motivation and school belonging in first-generation learners’, *KMAN Counseling & Psychology Nexus* 3, 1–10. 10.61838/kman.ec.psynexus.3.7

[CIT0044] Tshiloane, K.B. & Sefotho, M., 2025, ‘Experiences of teachers implementing inclusive education in full-service schools’, *African Journal of Teacher Education and Development* 4(1), a88. 10.4102/ajoted.v4i1.88

[CIT0045] UNESCO, 1994, ‘Salamanca Statement’, *The salamanca statement and framework for action on special needs education*, viewed n.d., from https://unesdoc.unesco.org/ark:/48223/pf0000098427.

[CIT0046] UNESCO, 2009, *UNESCO framework for cultural statistics*, viewed 07 November 2025, from https://unesdoc.unesco.org/ark:/48223/pf0000191061?form=MG0AV3.

[CIT0047] Vincent, C., 2019, *Nancy fraser, social justice and education*, Routledge, London.

[CIT0048] Walton, E., 2018, ‘Decolonising (through) inclusive education?’, *Educational Research for Social Change* 7, 31–45. 10.17159/2221-4070/2018/v7i0a3

[CIT0049] Yin, N., 2018, ‘The influencing outcomes of job engagement: An interpretation from the social exchange theory’, *International Journal of Productivity and Performance Management* 67(5), 873–889. 10.1108/IJPPM-03-2017-0054

